# Machine Learning-Based Identification of Suicidal Risk in Patients With Schizophrenia Using Multi-Level Resting-State fMRI Features

**DOI:** 10.3389/fnins.2020.605697

**Published:** 2021-01-11

**Authors:** Bartosz Bohaterewicz, Anna M. Sobczak, Igor Podolak, Bartosz Wójcik, Dagmara Mȩtel, Adrian A. Chrobak, Magdalena Fa̧frowicz, Marcin Siwek, Dominika Dudek, Tadeusz Marek

**Affiliations:** ^1^Department of Cognitive Neuroscience and Neuroergonomics, Institute of Applied Psychology, Jagiellonian University, Kraków, Poland; ^2^Department of Psychology of Individual Differences, Psychological Diagnosis, and Psychometrics, Institute of Psychology, University of Social Sciences and Humanities, Warsaw, Poland; ^3^Institute of Computer Science, Faculty of Mathematics and Computer Science, Jagiellonian University, Kraków, Poland; ^4^Department of Community Psychiatry, Jagiellonian University Medical College, Kraków, Poland; ^5^Department of Adult Psychiatry, Jagiellonian University Medical College, Kraków, Poland; ^6^Department of Affective Disorders, Jagiellonian University Medical College, Kraków, Poland

**Keywords:** schizophrenia, suicidal ideations, machine learning, resting state fMRI, mental pain, classification, gradient boosting, feature selection

## Abstract

**Background:**

Some studies suggest that as much as 40% of all causes of death in a group of patients with schizophrenia can be attributed to suicides and compared with the general population, patients with schizophrenia have an 8.5-fold greater suicide risk (SR). There is a vital need for accurate and reliable methods to predict the SR among patients with schizophrenia based on biological measures. However, it is unknown whether the suicidal risk in schizophrenia can be related to alterations in spontaneous brain activity, or if the resting-state functional magnetic resonance imaging (rsfMRI) measures can be used alongside machine learning (ML) algorithms in order to identify patients with SR.

**Methods:**

Fifty-nine participants including patients with schizophrenia with and without SR as well as age and gender-matched healthy underwent 13 min resting-state functional magnetic resonance imaging. Both static and dynamic indexes of the amplitude of low-frequency fluctuation (ALFF), the fractional amplitude of low-frequency fluctuations (fALFF), regional homogeneity as well as functional connectivity (FC) were calculated and used as an input for five machine learning algorithms: Gradient boosting (GB), LASSO, Logistic Regression (LR), Random Forest and Support Vector Machine.

**Results:**

All groups revealed different intra-network functional connectivity in ventral DMN and anterior SN. The best performance was reached for the LASSO applied to FC with an accuracy of 70% and AUROC of 0.76 (*p* < 0.05). Significant classification ability was also reached for GB and LR using fALFF and ALFF measures.

**Conclusion:**

Our findings suggest that SR in schizophrenia can be seen on the level of DMN and SN functional connectivity alterations. ML algorithms were able to significantly differentiate SR patients. Our results could be useful in developing neuromarkers of SR in schizophrenia based on non-invasive rsfMRI.

## Introduction

Schizophrenia research suggests that as much as 40% of all death causes in this group can be attributed to suicides (Wildgust et al., 2010), while 25–50% of individuals with schizophrenia attempt to commit suicide during their lifetime (Bohaterewicz et al., 2018; Cassidy et al., 2018). Hence, there is a vital need of developing more accurate and objective methods to predict the risk of suicide among individuals with schizophrenia.

Functional magnetic resonance imaging (fMRI) is a non-invasive, widely employed method allowing one to measure activity of a human brain. Resting state (rs), in turn, is considered highly effective as it captures 60–80% of the brain’s total activity (Smitha et al., 2017). Furthermore, some studies show that it allows monitoring treatment outcomes as well as assessing biomarkers of psychiatric disorders (Glover, 2011; Moghimi et al., 2018).

Previous studies indicate gray matter volume reduction in dorsolateral prefrontal cortex (DLPFC), superior temporal gyrus, as well as insular cortex in patients after suicide attempt, compared to the ones without suicide attempt in the past (Besteher et al., 2016; Zhang et al., 2020), whereas fMRI studies revealed that during a simple task based on cognitive control, suicide thoughts were associated with decreased activity in PFC and the history of previous suicide attempt resulted in decreased activity of premotor cortex (Minzenberg et al., 2014; Potvin et al., 2018). Previous results from volumetric as well as functional task fMRI analyses indicate the potential resting-state brain activity changes in the regions included in Default Mode Network (DMN), Salience Network (SN), and Sensorimotor Network (SMN).

In recent years, there has been a growing number of machine learning (ML) applications on rsfMRI data in order to make prognostic evaluation and to differentiate between various groups or conditions (Pereira et al., 2009). Lately, ML classifiers with the input from fMRI as an unbiased biomarker have been adopted to identify people engaged in the suicide-related behavior, including suicide ideations. For example, Just et al. (2017) were able to correctly identify 15 out of 17 suicidal participants with a sensitivity of 0.88 and a specificity of 0.94 using Gaussian Naïve Bayes algorithm and task fMRI data. In more recent work, Gosnell et al. (2019) used a Random Forest (RF) algorithm and rsfMRI functional connectivity data from psychiatric inpatients that enabled them to correctly classify suicidal behavior with a sensitivity of 81.3%. To the best of our knowledge, none of the previous studies focused on various ML classifiers in order to discriminate between healthy controls (HCs), suicidal risk (SR), and non-suicidal risk (NSR) schizophrenia patients based on rsfMRI data.

In the current work, our objective was to conjoin ML methods with rsfMRI data in order to investigate whether the selected classifiers allow differentiating between schizophrenia patients with and without a suicide risk. Ultimately, five algorithms such as Gradient Boosting (GB), Least Absolute Shrinkage and Selection Operator (LASSO), Logistic Regression (LR), RF, and Support Vector Machine (SVM) were performed to increase the reliability of diagnostic accuracy. Each metric presents a different degree of complexity; therefore, establishing their separate and combined precision allows gaining a wider picture in the aforementioned classification. Moreover, the article aims at explaining the association between the resting-state brain activity and suicide risk among schizophrenia patients; thus, stationary as well as dynamic measures with sliding windows approach were calculated. Based on the literature, the authors hypothesize (a) varied neural activity in the regions included in DMN, SN, and SMN networks to be involved in suicide risk in patients with schizophrenia; (b) that the predictive ability of classifiers will be better while using dynamic indexes as the features in comparison to the static ones; (c) that the results of ML-based discrimination will differ using diverse parcellation approaches; (d) ML-based algorithms to discriminate between SR and NSR groups with the accuracy exceeding 50%.

## Materials and Methods

### Participants

A total of 66 participants were recruited for the study. The clinical group consisted of 43 patients with paranoid schizophrenia diagnosed according to the ICD-10 criteria. The clinical group covered two subgroups: 24 patients with acute suicidal risk (SR) and 19 patients without such risk (NSR). The control group (HCs) consisted of 23 individuals matched in terms of gender and age with a clinical group, without diagnosis of mental disorder or a history of mental illness in first-degree relatives. All the participants were right-handed, as measured by the Neurological Evaluation Scale (Buchanan and Heinrichs, 1989). The inclusion criterion for the clinical group was treatment with atypical antipsychotic agents from the group of dibenzoxazepine: clozapine, olanzapine, or quetiapine. Additionally, valproic acid treatment was accepted. The exclusion criteria for both clinical and control groups were as follows: (1) history of alcohol or drug abuse (according to substance use disorder of DSM-5); (2) severe, acute, or chronic neurological and somatic diseases; (3) severe personality disorders; (4) treatment other than those mentioned in the inclusion criteria. All of the abovementioned conditions were confirmed by clinical interviews based on DSM-5 criteria. Detailed information about patients’ medication is presented in the Supplementary Materials. Written consent was obtained from all of the participants. The study was approved by the Jagiellonian University Bioethics Committee.

### Assessment of Suicidality

Suicidal risk was assessed with the Polish adaptation of Suicide Behavior Questionnaire—Revised (SBQ-R) (Osman et al., 2001; Chodkiewicz and Gruszczyńska, 2020), with the cutoff of ≥8 points in accordance with the Osman et al. (2001) recommendations. Moreover, the Polish adaptation (Chodkiewicz, 2013) of The Psychache Scale (TPS) (Holden et al., 2001) was used to evaluate the subjective experience of participants’ psychological pain, considered to be highly associated with suicidal thoughts and acts (Ducasse et al., 2018).

### MRI Data Acquisition

MRI data were acquired using a 3-T Siemens Skyra MR System (Siemens Medical Solutions, Erlangen, Germany). Anatomical images were obtained using sagittal 3D T1-weighted MPRAGE sequence with TR = 2,300 ms and TE = 3.9 ms. A total of 13 min of functional resting-state BOLD images was acquired using a gradient-echo single-shot echo planar imaging sequence with the following parameters: FOV = 256 mm; TE = 27 ms; TR = 2060 ms; voxel size = 3 mm × 3 mm × 3 mm, with no gap. Altogether, 39 interleaved transverse slices and 400 volumes were acquired. During the resting-state procedure, the subjects were instructed to keep their eyes open and to think of nothing particular. They were also asked not to fall asleep, which was controlled using an infrared binocular eye tracker (Eyelink 1000 Plus, SR Research, Mississauga, ON, Canada). In addition, during the EPI sequence, neutral gray background was presented using a MRI-compatible LCD screen and Siemens Head Coil Viewing Mirror. Both structural and functional sequence details are in Supplementary Data Sheet 1.

### Data Preprocessing

Data preprocessing was performed using Dpabi v. 4.2 (Yan et al., 2016) and SPM 12 (Friston, 1994), both working under Matlab v.2018a (The Mathworks Inc.). The first 10 time points were discarded due to signal equilibration, and next slice timing and realignment with assessment of the voxel specific head motion were conducted. The subjects with movements in one or more of the orthogonal directions above 3 mm or rotation above 3° were discarded from the analysis. A total of three participants from the control group and four patients from the clinical sample (four from SR group) were excluded due to the excessive head movements. Consequently, 39 patients and 20 HCs were included in the final analyses. Functional scans were then coregistered using T1 images and normalized to Montreal Neurological Institute (MNI) space using DARTEL and a voxel size of 3 mm^3^. The 24 motion parameters (Friston et al., 1996) derived from the realignment step were regressed out from the functional data by linear regression as well as five principal components from both cerebrospinal fluid and white matter signals using principal components analysis integrated in a Component-Based Noise Correction Method (Behzadi et al., 2007). The global signal was included due to its potential in providing additional valuable information (Liu et al., 2017). The signal was then band-pass filtered (0.01–0.08 Hz). Finally, the functional data were spatially smoothed with 4-mm Full Width at Half Maximum (FWHM) kernel.

### Parcellation

For validation purposes and to exclude a chance of parcellation-specific results, the preprocessed data were parcellated using two functional atlases: Power et al. (2011), which utilizes 264 functionally independent regions, and Automated Anatomical Labeling (AAL) atlas, which separates brain into 116 regions (Tzourio-Mazoyer et al., 2002). Using centroids obtained from both atlases, the raw signal from individual brain maps was extracted and averaged within a 4-mm-radius sphere using MarsBaR v. 0.43 (Brett et al., 2002). In addition, in accordance to our hypothesis, in order to investigate possible between-group differences among DMN, SN, and SM networks, the authors used templates from FIND lab^1^. Raw time series were extracted and averaged in each ROI within ventral default mode network (vDMN), dorsal default mode network (dDMN), anterior salience network (aSN), posterior salience network (pSN), and SMN (Shirer et al., 2012) (see Supplementary Table 1 for detailed information about the ROIs).

### Measures

**FIGURE 1:**
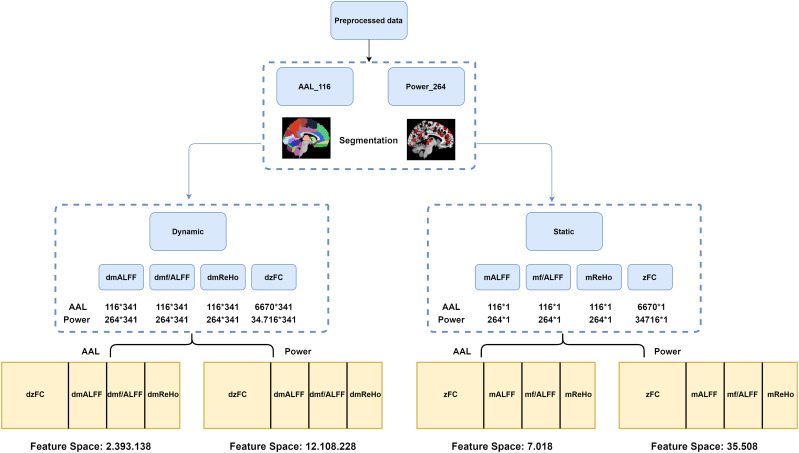
Flowchart of the steps taken in order to extract rsfMRI features.

For the purpose of developing a predictive classification model, the authors used Regional Homogeneity (ReHo), Amplitude of Low Frequency Fluctuations (ALFF), Fractional Amplitude of Low Frequency Fluctuations (fALFF), and Functional Connectivity (FC). Each measure has its static and dynamic equivalent, and each measure was extracted for both atlases (see Figure 1 for study flowchart and Supplementary Materials for detailed description of the measures).

### Stationary Approach

The mReHo, ALFF, and f/ALFF maps were segmented into 116 and 264 brain regions, and the values were extracted accordingly. Each participant got 116 × 1 (AAL atlas) and 264 × 1 (Power et al. atlas) matrices, consisting of a single value for each brain region among all 390 time points. Z-transformed correlation coefficients were obtained for each brain atlas, which resulted in 264 × 264 as well as 116 × 116 matrices for each participant. The lower half and diagonal values were removed and not used as features for ML algorithms, so that the total of 6,670 and 34,716 *z*-score values were used.

### Dynamic Approach

The dynamic mALFF, mf/ALFF, and mReHo indexes were computed using the Temporal Dynamic Analysis module of Dpabi (Yan et al., 2017) using a sliding window approach with a hamming window shape.

According to previous work of Leonardi and Van De Ville (2015), in order to reduce the likelihood of spurious fluctuations in the dynamics of observed data, minimum window length should have at least 1/*f*_*min*_, where *f*_*min*_ is the minimum frequency of the time series; a similar approach was used among others in the work of Li et al. (2019), where the authors showed alterations in temporal dynamics of the brain associated with suicidal ideations in depression. In our case, *f*_*min*_ after band-pass filter equals 0.01 Hz (100 s), and for this reason, window size was set to 50 TR (103 s) length and was shifted by 1 TR (2.06 s). The full time series was divided into 341 windows for each participant. As in case of stationary maps, dynamic maps were segmented using AAL and Power atlases. As a result, each participant gets a matrix of size 341 × 116 for AAL atlas and 341 × 264 for Power et al. atlas for dynamic ReHo (dReHo), dynamic ALFF (dALFF), as well as dynamic f/ALFF (df/ALFF). Each column represented a brain region and rows were populated with a single value for each window. Dynamic functional connectivity (dFC) was computed using the same window size, shift, and method via DynamicBC v.2.2 (Liao et al., 2014). After the calculations, data were represented by a 341 × 116 × 116 matrix for AAL atlas and 341 × 264 × 264 for Power atlas, where each of the 341 windows were “populated” with *z*-score Pearson correlation values. As in the case of stationary FC, upper half and diagonal values were removed from each of the 341 matrices so that 6670 and 34,716 *z*-score values for each window were used, which gave a total of 2.274470 (aal) and 11.838156 (Power) *z*-score values used as an input for classification algorithms.

### Classification Models

The authors used a selection of the most effective classification algorithms, each with a different level of complexity: LR (Cramer, 2002), LASSO (Tibshirani, 1996), SVM (Boser et al., 1992), RF (Ho, 1995), and GB (Friedman, 2001). See Supplementary Materials for a detailed description of the algorithms. Python, SciPy, NumPy, and scikit-learn (ver. 0.21) (Pedregosa et al., 2011) were used to compute the results. Standard scikit-learn model classes, score calculation routines, grid search, and dataset splitting functions were applied where possible.

The source code is available at https://github.com/gmum/schizo_fmri.

### Classification Framework

**FIGURE 2:**
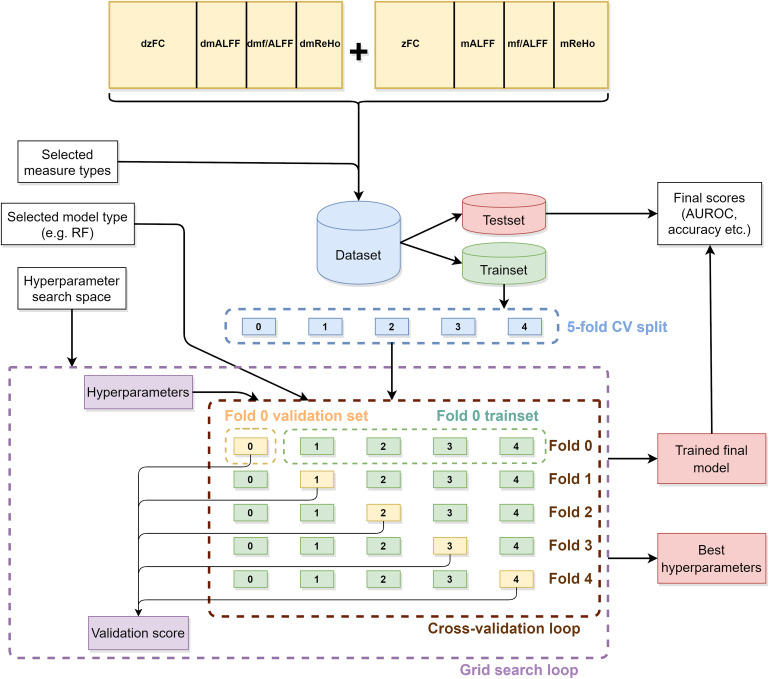
Illustration of the machine learning classification framework.

The dataset was divided into train and test sets of approximately equal sizes (19 and 20 patients, respectively) with stratification. The training dataset was used to train a classifier pipeline consisting of optional data standardization and dimensionality reduction using Principal Component Analysis (PCA) steps and of the actual classifier. A grid search with fivefold cross-validation was performed to find the optimal hyperparameters. The entire hyperparameter grid search and training procedure is illustrated in Figure 2. It was run separately for every combination of the classifier type, input data type, and whether dimensionality reduction was performed.

For static data, the model simply accepts the entire data of selected measure types for the given patient. For dynamic data, the model is given only a single window and, thus, predicts a binary label for each window. An aggregation scheme is needed to make a final prediction for a patient. For this purpose, we applied a simple thresholding and counting scheme. Window results are first transformed to binary results using 0.5 as the threshold value. The final result was defined as the ratio of positive results to the total number of windows.

### Classification Performance

Area Under Receiver Operating Curve (AUROC) was used as the score metric for grid search cross-validation procedure, and both AUROC and accuracy of the final classifier were evaluated on the test dataset.

In addition, the *p*-value of each AUROC score was calculated in order to determine the statistical significance of the obtained results. To do this, 1,000 permutations of the target labels were generated. Then, for each permutation, the classifier was retrained on the permuted labels, and its AUROC score was measured. The *p*-value was defined as the ratio of runs that achieved a score greater than or equal to the score of the original classifier (Ojala and Garriga, 2010).

### Statistical Analysis

Two-sample *t*-tests were used in order to seek for possible differences in suicidal risk (SBQ-R), mental pain (TPS), severity, and illness duration between the SR and NSR groups. One-way analysis of variance (ANOVA) was used to check for possible differences in age, mean FD, and education (in years) between SR, NSR, and HCs groups, and the Kruskal–Wallis *H* test was used to compare gender differences.

One-way ANOVA was used to investigate possible differences in FC among three rsfMRI networks between SR, NSR, and HCs groups. The results were corrected with the Benjamini and Hochberg (1995) False Discovery Rate correction at *p* < 0.05.

One-way ANCOVA with the Bonferroni *post hoc* test was used to investigate the differences in static ALFF, fALFF, ReHo between SR, NSR, and HCs. Age, gender, and mean FD were used as covariates. The same set of analyses was applied to compare the group-level temporal variability of ALFF, fALFF, ReHo, and FC. Temporal variability for ALFF, fALFF, and ReHo was expressed as a coefficient of variations (SD/mean) and, for FC, as a variance calculated across sliding-window dynamics, and then compared using one-way ANOVA with FDR correction. The statistical significance level for ALFF, fALFF, and ReHo analyses was set as *p*_*FWER*_ < 0.05 with 5,000 times permutation using Permutation Analysis of Linear Models (Winkler et al., 2016) as a part of DPABI. The cluster forming threshold was set to *z* = 2.3, which is equal to *p* < 0.01 and the cluster extent threshold at *k* > 25 (Li et al., 2019).

## Results

### Demographic and Clinical Characteristics

The final analysis was conducted based on the data obtained from 59 participants, 39 of whom were schizophrenia patients. No differences in age [one-way ANOVA; *F*_(2,57)_ = 2.13; *p* = 0.1282], gender [Kruskal–Wallis; *H*_(2)_ = 2,468; *p* = 0.291], and head motion [one-way ANOVA; *F*_(2,57)_ = 0.66; *p* = 0.5214] were found among the three groups. SR and NSR groups were significantly different in SBQ-R score (*t* = 7.645; *p* < 0.001) and illness duration (*t* = 1.69; *p* = 0.01), but no differences were found in the case of TPS scores (*t* = 1.904; *p* = 0.064) The range of SBQ-R score in the SR group was 8–17 points (Table 1).

**TABLE 1:** Detailed participant demographic and clinical information.

**Demographics**	**HCs**	**SR**	**NSR**
Group size (*n*)	20	20	19
FD (0–3)	0.079 ± 0.04	0.09 ± 0.05	0.097 ± 0.063
TPS (13–59)	15.38 ± 6.88	33.65 ± 10.24	26.57 ± 12.86
SBQ-R (3–17)	4.42 ± 2.11	10.7 ± 2.97	5.10 ± 1.32
Gender (female/male)	10/10	5/15	9/10
Age (27–65)	36.57 ± 7.25	42.6 ± 9.4	39.1 ± 9.23
Handedness (right/left)	20/0	20/0	19/0
Illness duration (years) (10–39)	–	18 ± 10.1	10.89 ± 5.93

*SBQ-R, Suicide Behaviors Questionnaire—Revised; TPS, The Psychache Scale. Ranges of the variables are provided in the parentheses.*

### Differences in rsfMRI Measures

**FIGURE 3:**
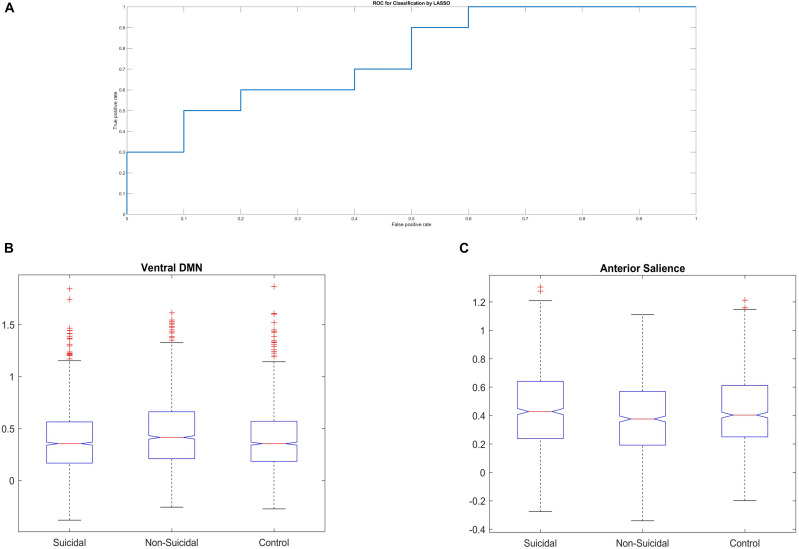
**(A)** Receiver operating characteristic curve for LASSO classificator applied to Functional Connectivity with Power 264 atlas. **(B)** Box-and-whisker plot illustrating the differences between three groups in Functional Connectivity among ventral Default Mode Network. **(C)** Box-and-whisker plot illustrating the differences between three groups in Functional Connectivity among Anterior Salience Network. The outliers can be seen due to the high variance of dFC values.

No differences were found among the three groups in static ALFF, fALFF, and ReHo. No significant differences were found in the case of temporal variability of ALFF, fALFF, or ReHo either. Significant differences between SR, NSR, and HCs groups were found in both static functional connectivity and temporal variability of FC. One-way ANOVA showed that the three groups were different in FC among ventral DMN (*F* = 19.02; *p* < 0.001) and anterior SN (*F* = 6.85; *p* = 0.001) (Figure 3). *Post hoc* tests showed that the significant differences among ventral DMN network were present between SR and NSR groups (*p* < 0.001; FDR corrected) and NSR and HCs groups (*p* < 0.01; FDR corrected). In the case of anterior SN, *post hoc* tests indicated differences between SR and NSR groups (*p* = 0.03; FDR corrected). No differences were found among dorsal DMN, posterior SN, or SMN between the three groups. Temporal variability of FC, calculated at each voxel for the two atlases, showed that the three groups were significantly (*p* < 0.001) different in total FC variability measured using AAL, as well as Power atlases. *Post hoc* tests with FDR correction showed that in the case of both atlases, FC variability was significantly different between SR and NSR, between SR and HCs, and between NSR and HCs groups (Table 2 and Supplementary Figures 1,2).

**TABLE 2:** Dynamic functional connectivity and functional connectivity differences among NSR, SR, and HC groups.

**Region**	***F***	***p***	**Pairwise comparison**
Dorsal DMN	1.55	0.2133	–
Ventral DMN	19.02	<0.001	SR vs. NSR (*p* < 0.0001, MD = −0.0582); NSR vs. HCs (*p* < 0.0001, MD = 0.0550)
Anterior SN	6.85	0.0011	SR vs. NSR (*p* = 0.001, MD = 0.0482); NSR vs. HCs (*p* = 0.029, MD = −0.0347)
Posterior SN	1.23	0.2919	–
Sensorimotor Network	1.66	0.1898	–
Dynamic variance *Power Atlas* FC	677.67	<0.001	SR vs. NSR (*p* < 0.0001, MD = −0.00103); SR vs. HCs (*p* < 0.0001, MD = −0.00095); NSR vs. HCs (*p* = 0.0404, MD = 0.0000775)
Dynamic variance *AAL* FC	108.61	<0.001	SR vs. NSR (*p* < 0.0001, MD = −0.00098); SR vs. HCs (*p* < 0.0001, MD = −0.00068); NSR vs. HCs (*p* = 0.00031, MD = 0.000303)

*Statistical differences were computed using one-way ANOVA with FDR correction.*

### Classification Results

The 10 most important features of amplitude of low-frequency fluctuations (ALFF) for LR as well as of Functional Connectivity for LASSO classifiers are presented in Table 3. The accuracies and AUROCs of five ML algorithms for each static and dynamic rsfMRI measures, divided into two atlases, are listed in Table 4.

**TABLE 3:** The 10 most important features for Logistic Regression and LASSO classifiers.

**Ensemble Method**	**Logistic Regression**	
**Feature**	**ROI labels**	**Coordinates (MNI)**
1	Angular_L	−39, −74, 43
2	Precuneus_L	−11, −56, 15
3	Cingulum_Ant_L	−2, 41, 16
4	Temporal_Sup_L	−60, −25, 13
5	Postcentral_R	65, −7, 24
6	Precentral_R	20, −29, 60
7	Postcentral_R	50, −20, 42
8	Postcentral_L	−53, −10, 24
9	Rolandic_Oper_R	43, −23, 20
10	Frontal_Sup_L	−20, 45, 39

**Ensemble Method**	**LASSO**	
**Feature**	**ROI–ROI labels**	

1	Supramarginal_R–Precentral_R	
2	Postcentral_R–Precuneus_R	
3	Angular_R–Precentral_R	
4	Frontal_Sup_Medial_R–Parietal_Sup_R	
5	Paracentral_Lobule_L–Postcentral_R	
6	Frontal_Sup_Medial_R–Frontal_Mid_R	
7	Precentral_R–Frontal_Sup_Medial_R	
8	Frontal_Inf_Tri_L–Occipital_Mid_L	
9	Precentral_R–Frontal_Sup_Medial_R	
10	Frontal_Sup_Medial_L–Precentral_R	

*Data are presented for (a) Logistic Regression applied to dynamic amplitude of low-frequency fluctuations with AAL atlas and (b) LASSO applied to static ROI–ROI Functional Connectivity with Power 264 atlas.*

**TABLE 4:** The accuracies and AUROCs of five ML algorithms for each static and dynamic rsfMRI measures, divided into two atlases.

	**ALFF**		**fALFF**		**FC**		**REHO**		**Concatenated**	
**AAL 116 Static**	**With PCA**	**Without PCA**	**With PCA**	**Without PCA**	**With PCA**	**Without PCA**	**With PCA**	**Without PCA**	**With PCA**	**Without PCA**
GB	55%/0.6	55%/0.62	65%/0.74*	40%/0.25	65%/0.65	55%/0.47	45%/0.46	45%/0.43	55%/0.62	55%/0.49
LASSO	55%/0.41	50%/0.41	55%/0.54	65%/0.61	50%/0.58	50%/0.65	65%/0.54	50%/0.6	50%/0.27	45%/0.55
LR	60%/0.55	45%/0.5	35%/0.29	35%/0.34	35%/0.34	35%/0.32	35%/0.36	35%/0.32	45%/0.43	35%/0.29
RF	45%/0.57	50%/0.55	65%/0.66	30%/0.34	50%/0.43	60%/0.44	30%/0.32	25%/0.24	55%/0.67	50%/0.36
SVM	50%/0.46	50%/0.42	50%/0.55	40%/0.29	50%/0.14	40%/0.37	50%/0.38	30%/0.34	45%/0.38	60%/0.48
**AAL 116 Dynamic**										
GB	45%/0.47	60%/0.5	40%/0.42	50%/0.5	–	–	60%/0.5	40%/0.29	–	–
LASSO	40%/0.45	50%/0.39	60%/0.57	50%/0.45	–	–	40%/0.43	55%/0.49	–	–
LR	50%/0.65	65%/0.75*	60%/0.56	35%/0.39	35%/0.27	–	50%/0.51	35%/0.41	45%/0.44	–
RF	45%/0.49	50%/0.57	35%/0.48	45%/0.47	50%/0.43	–	35%/0.38	40%/0.41	70%/0.6	–
SVM	45%/0.46	45%/0.42	40%/0.42	40%/0.41	–	–	45%/0.32	55%/0.39	–	–
Power 264 Static										
GB	60%/0.55	40%/0.5	40%/0.32	55%/0.56	45%/0.67	50%/0.5	40%/0.4	30%/0.33	50%/0.62	50%/0.5
LASSO	45%/0.42	25%/0.42	70%/0.65	55%/0.31	50%/0.41	**70%/0.76**	35%/0.34	35%/0.36	45%/0.51	50%/0.41
LR	55%/0.61	60%/0.48	45%/0.51	40%/0.44	45%/0.47	45%/0.43	50%/0.51	40%/0.45	45%/0.48	45%/0.4
RF	60%/0.65	40%/0.41	45%/0.47	55%/0.46	60%/0.69	55%/0.38	55%/0.68	70%/0.57	55%/0.48	50%/0.49
SVM	50%/0.34	50%/0.39	50%/0.45	50%/0.33	50%/0.68	50%/0.46	45%/0.24	40%/0.43	50%/0.69	50%/0.46
**Power 264 Dynamic**										
GB	55%/0.58	45%/0.6	50%/0.5	60%/0.57	–	–	55%/0.6	55%/0.64	–	–
LASSO	60%/0.5	55%/0.65	45%/0.62	45%/0.38	–	–	45%/0.58	45%/0.5	–	–
LR	60%/0.57	70%/0.66	55%/0.51	50%/0.44	50%/0.46	–	50%/0.47	50%/0.5	50%/0.48	–
RF	45%/0.44	55%/0.53	50%/0.34	50%/0.48	50%/0.41	–	40%/0.61	60%/0.55	50%/0.49	–
SVM	45%/0.46	45%/0.6	40%/0.47	55%/0.42	–	–	50%/0.55	50%/0.51	–	–

*GB, gradient boosting; LASSO, least absolute shrinkage and selection operator; LR, logistic regression; RF, random forest; SVM, support vector machine. Significant results (p < 0.05), estimated using the permutation method, are marked with an asterisk and bolded. The binary classification was conducted using NSR and SR groups.*

Three variants of ML algorithm and rsfMRI measures turned out to be significant at *p* < 0.05. (1) The LASSO applied to static functional connectivity with Power atlas reached an accuracy of 70% and an AUROC of 0.76. (2) The LR algorithm applied to dynamic ALFF with AAL atlas reached an accuracy of 65% and an AUROC of 0.75. (3) The GB algorithm applied to static fALFF with AAL atlas reached an accuracy of 65% and an AUROC of 0.74. It can be seen that AUROCs of the majority of variants were at chance level, even when accuracies were above 50%. In short, the obtained results suggest that the combination of LASSO algorithm and static functional connectivity calculated on 264 ROIs provide superior accuracy/AUROC of classification between SR patients and non-SR patients and allow correct classification of 14 out of 20 SR patients.

## Discussion

In the present article, a successful discrimination between schizophrenia patients with and without a suicide risk using ML algorithms and rsfMRI data was demonstrated.

Although previous studies developed the rsfMRI-based ML classification models capable of distinguishing suicidal patients with different diagnoses, such as anxiety disorder, depression, or borderline personality disorder (Gosnell et al., 2019; Wang et al., 2020), to the best of the authors’ knowledge, none of the previous studies were focused on schizophrenia. *Ipso facto*, the presented study is the first attempt to find rsfMRI features that allow detecting the risk of suicide in schizophrenia with the use of the ML algorithms. Moreover, this is the first work using rsfMRI to explain the differences in brain activity, which might be associated with suicide risk in schizophrenia patients. Above that, unlike most of ML–fMRI studies focused on classifying suicidal participants, the authors used five various ML classifiers. The results of conventional analyses showed that patients with and without suicidal risk, as well as the healthy controls, demonstrated different patterns of temporal variability of dFC and FC, with the latter being an important feature for ML classification. Furthermore, ALFF and fALFF measures also contributed to ML-based classification, but no significant differences in the above measures were found in the conventional group analyses.

Du et al. (2015) and also Gosnell et al. (2019) results are partly congruent with the ones obtained from this study, indicating frontal and temporal brain region abnormalities to be the most useful in ML classification of suicidal risk, suggesting activity of specific brain structures to be characteristic for suicide risk among all psychiatric patients. Cao et al. (2015), in turn, demonstrated altered ReHo in suicide attempters without psychiatric diagnosis in left precuneus, which also remains consistent with the region allowing discriminating between SR and NSR patients reported in our study. In addition, regions that turned out to be discriminative in ML-based classification are also congruent with the studies using cognitive control tasks, revealing the association between decreased activity of the frontal cortex and suicide risk in schizophrenia (Zhang et al., 2013; Potvin et al., 2018). Our results are also consistent with the studies showing significance of ACC, angular gyrus, as well as both precentral and postcentral gyrus in understanding the suicidal behavior (Reisch et al., 2010; Fan et al., 2013; Tsujii et al., 2017; Harms et al., 2019).

Numerous fMRI studies indicate the association of suicide-related behaviors and prefrontal cortex alterations due to its role in decision-making as well as action planning (Potvin et al., 2018; Brown et al., 2020). Other studies suggest decreased connectivity between ACC and PFC to be related to suicidal behavior (Minzenberg et al., 2015; Chase et al., 2017). PFC and ACC are considered responsible for anticipating the consequence of actions, inhibition of inappropriate behavior, and impulsiveness (Zhou et al., 2016; Brown et al., 2019), which are indirectly related to suicidal behavior (Wang et al., 2017; Koval and Baumann, 2019). Alterations in ACC and PFC have also been found in patients with schizophrenia (Cordes et al., 2015; Fryer et al., 2019) while their impulsivity has been reported as correlated with increased suicide risk (Iancu et al., 2010). Notably, the above regions are included in DMN as well as SN, which have already been established as disrupted in schizophrenia (Garrity et al., 2007; White et al., 2010; Palaniyappan et al., 2012). Weaker functional connectivity within DMN is reported to be associated with the difficulties in abstract thinking, planning the future, as well as analyzing social behaviors (Andrews-Hanna, 2012), while decreased functional connectivity within SN has been reported as linked to higher trait anxiety and decreased cognitive regulation (Geng et al., 2016). The above symptoms might additionally elevate suicidal risk.

Importantly, our results from conventional static FC analyses confirm the aforementioned results, revealing functional connectivity differences in ventral DMN and anterior SN between SR and NSR patients. The above dissimilarity was also apparent between NSR patients and HCs. Moreover, The LASSO algorithm, applied on static functional connectivity data, allowed the discrimination between SR and NSR patients, supporting our hypothesis. Furthermore, all the three groups varied from each other in the case of temporal variability of dFC with the use of both AAL and Power atlases.

According to another hypothesis, all five ML algorithms presented different classification performance. The LASSO, applied on static functional connectivity without PCA on Power atlas, achieved 70% accuracy and an AUROC of 0.76, proving to be the best ML classifier used in the study. As a result, LASSO allowed the correct classification of 14 out of 20 suicidal patients. The possible reason why the LASSO outperforms the other classifiers is the type of its regularization loss term. The processed dataset has high dimensionality but very few samples, which could possibly make the standard classifiers heavily overfit to the train set and therefore perform poorly on the test set. As a result, a regularization method needs to be used in order to reduce overfitting and increase generalization. The L1 cost used in LASSO has a property of much stronger parameters’ shrinkage due to its diagonal regularization contour, leading to a more sparse model.

Noteworthy, raw performance of the classifiers differed depending on the selected parcellation scheme. However, contrary to our assumptions, the dynamic measures did not improve the prediction ability of ML classifiers compared to static measures. As far as the authors are aware, none of the previous studies used ML-based classifiers to discriminate between SR and NSR schizophrenia patients. Further studies should consider to enlarge sample size in order to demonstrate the replicability of our study.

## Limitations

The conducted study has some limitations. Firstly, EPI sequence was introduced to participants after the structural scans (T1–MPRAGE) and not before them, which could influence the results. Secondly, results may possibly depend on the size of the smoothing kernel. Another noteworthy limitation of this study is the restricted sample size; thus, the presented results should be interpreted with caution. Further studies should consider extending the sample size by adding the control group to the input training data. Moreover, the specific window size as well as arbitrarily chosen atlases could influence the results. What is more, the high number of features may cause high susceptibility to any noise signal; therefore, distinctive features could be possibly different in another sample size, i.e., patients. Based on the literature, we have also decided to include the global signal, which is not always considered to be beneficial. Additionally, further studies should incorporate a semi-supervised approach with a pre-training phase using the data of HC.

## Data Availability Statement

The raw data supporting the conclusions of this article will be made available by the authors, without undue reservation, to any qualified researcher.

## Ethics Statement

The studies involving human participants were reviewed and approved by Jagiellonian University Bioethics Committee. The patients/participants provided their written informed consent to participate in this study.

## Author Contributions

BB: conceptualization, methodology, validation, formal analysis, investigation, writing – original draft, writing – review and editing, visualization, and project administration. AMS: formal analysis, investigation, writing – original draft, writing – review and editing, and visualization. IP: methodology, software, validation, writing – review and editing, and supervision. BW: software, formal analysis, writing – original draft, and visualization. DM: resources and writing – original draft. AC: investigation, resources, and funding acquisition. MF: writing – review and editing. MS: resources. DD: resources. TM: resources, writing – review and editing, supervision, and funding acquisition. All authors contributed to the article and approved the submitted version.

## Conflict of Interest

The authors declare that the research was conducted in the absence of any commercial or financial relationships that could be construed as a potential conflict of interest.

## Footnotes

1 http://findlab.stanford.edu/functional_ROIs.html
