# Supplementary material for: Machine Learning-Based Identification of Suicidal Risk in Patients With Schizophrenia Using Multi-Level Resting-State fMRI Features
Source: Front Neurosci. 2021 Jan 11;14:605697. doi: 10.3389/fnins.2020.605697 (PMC7829970; doi:10.3389/fnins.2020.605697)
Supplement: Supplementary file 1 [file Data_Sheet_1.PDF]

\\USER\PROJECTS\WERGENCJA\schizophrenia_project\haste_localizer
TA: 0:40 PM: ISO Voxel size: 1.7×1.7×6.0 mmPAT: 2 Rel. SNR: 1.00 : h

Properties

Prio recon	Off
Load images to viewer	On
Inline movie	Off
Auto store images	On
Load images to stamp segments	On
Load images to graphic segments	On
Auto open inline display	Off
Auto close inline display	Off
Start measurement without further preparation	On
Wait for user to start	Off
Start measurements	Single measurement

Routine

Slice group	1
Slices	10
Dist. factor	150 %
Position	L0.0 P0.0 H50.0 mm
Orientation	Transversal
Phase enc. dir.	A >> P
Slice group	2
Slices	10
Dist. factor	150 %
Position	L0.0 P30.0 H0.0 mm
Orientation	Coronal
Phase enc. dir.	R >> L
Slice group	3
Slices	10
Dist. factor	150 %
Position	Isocenter
Orientation	Sagittal
Phase enc. dir.	A >> P
AutoAlign	---
Phase oversampling	30 %
FoV read	430 mm
FoV phase	100.0 %
Slice thickness	6.0 mm
TR	1340.0 ms
TE	85 ms
Averages	1
Concatenations	1
Filter	Distortion Corr.(2D), Normalize, Elliptical filter
Coil elements	HE1-4;NE1,2

Contrast - Common

TR	1340.0 ms
TE	85 ms
MTC	Off
Magn. preparation	None
Flip angle	160 deg
Fat suppr.	None
Water suppr.	None
Restore magn.	Off

Contrast - Dynamic

Averages	1
Averaging mode	Long term
Reconstruction	Magnitude
Measurements	1
Multiple series	Each measurement

Resolution - Common

FoV read	430 mm
FoV phase	100.0 %
Slice thickness	6.0 mm
Base resolution	256
Phase resolution	70 %
Phase partial Fourier	5/8
Interpolation	Off

Resolution - iPAT

PAT mode	GRAPPA
Accel. factor PE	2
Ref. lines PE	24
Reference scan mode	Integrated

Resolution - Filter Image

Image Filter	Off
Distortion Corr.	On
Mode	2D
Unfiltered images	Off
Prescan Normalize	Off
Normalize	On
B1 filter	Off

Resolution - Filter Rawdata

Raw filter	Off
Elliptical filter	On

Geometry - Common

Slice group	1
Slices	10
Dist. factor	150 %
Position	L0.0 P0.0 H50.0 mm
Orientation	Transversal
Phase enc. dir.	A >> P
Slice group	2
Slices	10
Dist. factor	150 %
Position	L0.0 P30.0 H0.0 mm
Orientation	Coronal
Phase enc. dir.	R >> L
Slice group	3
Slices	10
Dist. factor	150 %
Position	Isocenter
Orientation	Sagittal
Phase enc. dir.	A >> P
FoV read	430 mm
FoV phase	100.0 %
Slice thickness	6.0 mm
TR	1340.0 ms
Multi-slice mode	Single shot
Series	Descending
Concatenations	1

Geometry - AutoAlign

Slice group	1
Position	L0.0 P0.0 H50.0 mm
Orientation	Transversal
Phase enc. dir.	A >> P
Slice group	2

Geometry - AutoAlign

Position	L0.0 P30.0 H0.0 mm
Orientation	Coronal
Phase enc. dir.	R >> L
Slice group	3
Position	Isocenter
Orientation	Sagittal
Phase enc. dir.	A >> P
AutoAlign	---
Initial Position	Isocenter
L	0.0 mm
P	0.0 mm
H	0.0 mm
Initial Rotation	0.00 deg
Initial Orientation	Transversal

Geometry - Saturation

Fat suppr.	None
Water suppr.	None
Restore magn.	Off
Special sat.	None

Geometry - Navigator

System - Miscellaneous

Positioning mode	ISO
Table position	H
Table position	0 mm
MSMA	S - C - T
Sagittal	R >> L
Coronal	A >> P
Transversal	H >> F
Coil Combine Mode	Adaptive Combine
Save uncombined	Off
Matrix Optimization	Off
AutoAlign	---
Coil Select Mode	On - AutoCoilSelect

System - Adjustments

B0 Shim mode	Tune up
B1 Shim mode	TrueForm
Adjust with body coil	Off
Confirm freq. adjustment	Off
Assume Dominant Fat	Off
Assume Silicone	Off
Adjustment Tolerance	Auto

System - Adjust Volume

Position	Isocenter
Orientation	Transversal
Rotation	0.00 deg
A >> P	263 mm
R >> L	350 mm
F >> H	350 mm
Reset	Off

System - Tx/Rx

Frequency 1H	123.228035 MHz
Correction factor	1
Gain	High
Img. Scale Cor.	1.000
Reset	Off
? Ref. amplitude 1H	0.000 V

Physio - Signal1

1st Signal/Mode	None
TR	1340.0 ms
Concatenations	1

Physio - Cardiac

Magn. preparation	None
Fat suppr.	None
Dark blood	Off
FoV read	430 mm
FoV phase	100.0 %
Phase resolution	70 %

Physio - PACE

Resp. control	Off
Concatenations	1

Inline - Common

Subtract	Off
Measurements	1
StdDev	Off
Save original images	On

Inline - MIP

MIP-Sag	Off
MIP-Cor	Off
MIP-Tra	Off
MIP-Time	Off
Save original images	On

Inline - Composing

Inline Composing	Off
Distortion Corr.	On
Mode	2D
Unfiltered images	Off

Sequence - Part 1

Introduction	Off
Dimension	2D
Contrasts	1
Flow comp.	No
Multi-slice mode	Single shot
Echo spacing	4.46 ms
Bandwidth	476 Hz/Px

Sequence - Part 2

RF pulse type	Fast
Gradient mode	Fast
Hyperecho	Off
Turbo factor	179

Sequence - Assistant

Mode	Min flip angle
Min flip angle	140 deg
Allowed delay	30 s

\\USER\PROJECTS\WERGENCJA\schizophrenia_project\anat
TA: 5:12 PM: REF Voxel size: 1.0×1.0×1.1 mmPAT: 2 Rel. SNR: 1.00 : tfl

Properties

Prio recon	Off
Load images to viewer	On
Inline movie	Off
Auto store images	On
Load images to stamp segments	Off
Load images to graphic segments	Off
Auto open inline display	Off
Auto close inline display	Off
Start measurement without further preparation	Off
Wait for user to start	Off
Start measurements	Single measurement

Routine

Slab group	1
Slabs	1
Dist. factor	50 %
Position	L1.6 P14.3 F23.9 mm
Orientation	Sagittal
Phase enc. dir.	A >> P
AutoAlign	Head > Basis
Phase oversampling	0 %
Slice oversampling	0.0 %
Slices per slab	176
FoV read	256 mm
FoV phase	96.9 %
Slice thickness	1.10 mm
TR	2300.0 ms
TE	2.98 ms
Averages	1
Concatenations	1
Filter	Prescan Normalize
Coil elements	HE1-4

Contrast - Common

TR	2300.0 ms
TE	2.98 ms
Magn. preparation	Non-sel. IR
TI	900 ms
Flip angle	9 deg
Fat suppr.	None
Water suppr.	None

Contrast - Dynamic

Averages	1
Averaging mode	Long term
Reconstruction	Magnitude
Measurements	1
Multiple series	Off

Resolution - Common

FoV read	256 mm
FoV phase	96.9 %
Slice thickness	1.10 mm
Base resolution	256
Phase resolution	100 %
Slice resolution	100 %
Phase partial Fourier	Off
Slice partial Fourier	Off
Interpolation	Off

Resolution - iPAT

PAT mode	GRAPPA
Accel. factor PE	2
Ref. lines PE	24
Accel. factor 3D	1
Reference scan mode	Integrated

Resolution - Filter Image

Image Filter	Off
Distortion Corr.	Off
Prescan Normalize	On
Unfiltered images	Off
Normalize	Off
B1 filter	Off

Resolution - Filter Rawdata

Raw filter	Off
Elliptical filter	Off

Geometry - Common

Slab group	1
Slabs	1
Dist. factor	50 %
Position	L1.6 P14.3 F23.9 mm
Orientation	Sagittal
Phase enc. dir.	A >> P
Slice oversampling	0.0 %
Slices per slab	176
FoV read	256 mm
FoV phase	96.9 %
Slice thickness	1.10 mm
TR	2300.0 ms
Multi-slice mode	Single shot
Series	Interleaved
Concatenations	1

Geometry - AutoAlign

Slab group	1
Position	L1.6 P14.3 F23.9 mm
Orientation	Sagittal
Phase enc. dir.	A >> P
AutoAlign	Head > Basis
Initial Position	Isocenter
L	0.0 mm
P	0.0 mm
H	0.0 mm
Initial Rotation	0.00 deg
Initial Orientation	Transversal

Geometry - Navigator

System - Miscellaneous

Positioning mode	REF
Table position	H
Table position	0 mm
MSMA	S - C - T
Sagittal	R >> L
Coronal	A >> P
Transversal	F >> H
Coil Combine Mode	Adaptive Combine
Save uncombined	Off

System - Miscellaneous

Matrix Optimization	Off
AutoAlign	Head > Basis
Coil Select Mode	Off - AutoCoilSelect

System - Adjustments

B0 Shim mode	Standard
B1 Shim mode	TrueForm
Adjust with body coil	Off
Confirm freq. adjustment	Off
Assume Dominant Fat	Off
Assume Silicone	Off
Adjustment Tolerance	Auto

System - Adjust Volume

Position	L1.6 P14.3 F23.9 mm
Orientation	Sagittal
Rotation	0.00 deg
A >> P	248 mm
F >> H	256 mm
R >> L	194 mm
Reset	Off

System - Tx/Rx

Frequency 1H	123.228035 MHz
Correction factor	1
Gain	Low
Img. Scale Cor.	1.000
Reset	Off
? Ref. amplitude 1H	0.000 V

Physio - Signal1

1st Signal/Mode	None
TR	2300.0 ms
Concatenations	1

Physio - Cardiac

Magn. preparation	Non-sel. IR
TI	900 ms
Fat suppr.	None
Dark blood	Off
FoV read	256 mm
FoV phase	96.9 %
Phase resolution	100 %

Physio - PACE

Resp. control	Off
Concatenations	1

Inline - Common

Subtract	Off
Measurements	1
StdDev	Off
Save original images	On

Inline - MIP

MIP-Sag	Off
MIP-Cor	Off
MIP-Tra	Off
MIP-Time	Off
Save original images	On

Inline - Composing

Inline Composing	Off

Inline - Composing

Distortion Corr.	Off

Sequence - Part 1

Introduction	On
Dimension	3D
Elliptical scanning	Off
Reordering	Linear
Asymmetric echo	Off
Flow comp.	No
Multi-slice mode	Single shot
Echo spacing	7.1 ms
Bandwidth	240 Hz/Px

Sequence - Part 2

RF pulse type	Fast
Gradient mode	Normal
Excitation	Non-sel.
RF spoiling	On
Incr. Gradient spoiling	Off
Turbo factor	176

Sequence - Assistant

Mode	Off

\\USER\PROJECTS\WERGENCJA\schizophrenia_project\rs-fMRI
TA: 13:52 PM: FIX Voxel size: 3.0×3.0×3.0 mmPAT: 2 Rel. SNR: 1.00 : epfid

Properties

Prio recon	Off
Load images to viewer	On
Inline movie	Off
Auto store images	On
Load images to stamp segments	Off
Load images to graphic segments	Off
Auto open inline display	Off
Auto close inline display	Off
Start measurement without further preparation	Off
Wait for user to start	On
Start measurements	Single measurement

Routine

Slice group	1
Slices	39
Dist. factor	15 %
Position	L1.0 P2.6 F11.6 mm
Orientation	Transversal
Phase enc. dir.	A >> P
AutoAlign	Head > Brain
Phase oversampling	0 %
FoV read	192 mm
FoV phase	100.0 %
Slice thickness	3.0 mm
TR	2060 ms
TE	27.0 ms
Averages	1
Concatenations	1
Filter	Prescan Normalize
Coil elements	HE1-4

Contrast - Common

TR	2060 ms
TE	27.0 ms
MTC	Off
Flip angle	73 deg
Fat suppr.	Fat sat.

Contrast - Dynamic

Averages	1
Averaging mode	Long term
Reconstruction	Magnitude
Measurements	400
Delay in TR	0 ms
Multiple series	Off

Resolution - Common

FoV read	192 mm
FoV phase	100.0 %
Slice thickness	3.0 mm
Base resolution	64
Phase resolution	100 %
Phase partial Fourier	Off
Interpolation	Off

Resolution - iPAT

Accel. mode	GRAPPA
Accel. factor PE	2
Ref. lines PE	32

Resolution - iPAT

Reference scan mode	EPI/separate

Resolution - Filter Image

Distortion Corr.	Off
Prescan Normalize	On

Resolution - Filter Rawdata

Raw filter	Off
Elliptical filter	Off
Hamming	Off

Geometry - Common

Slice group	1
Slices	39
Dist. factor	15 %
Position	L1.0 P2.6 F11.6 mm
Orientation	Transversal
Phase enc. dir.	A >> P
FoV read	192 mm
FoV phase	100.0 %
Slice thickness	3.0 mm
TR	2060 ms
Multi-slice mode	Interleaved
Series	Interleaved
Concatenations	1

Geometry - AutoAlign

Slice group	1
Position	L1.0 P2.6 F11.6 mm
Orientation	Transversal
Phase enc. dir.	A >> P
AutoAlign	Head > Brain
Initial Position	Isocenter
L	0.0 mm
P	0.0 mm
H	0.0 mm
Initial Rotation	0.00 deg
Initial Orientation	Transversal

Geometry - Saturation

Fat suppr.	Fat sat.
Special sat.	None

System - Miscellaneous

Positioning mode	FIX
Table position	H
Table position	0 mm
MSMA	S - C - T
Sagittal	R >> L
Coronal	A >> P
Transversal	F >> H
Coil Combine Mode	Adaptive Combine
Matrix Optimization	Performance
AutoAlign	Head > Brain
Coil Select Mode	On - AutoCoilSelect

System - Adjustments

B0 Shim mode	Advanced
B1 Shim mode	TrueForm
Adjust with body coil	Off

System - Adjustments

Confirm freq. adjustment	Off
Assume Dominant Fat	Off
Assume Silicone	Off
Adjustment Tolerance	Auto

System - Adjust Volume

Position	L1.0 P2.6 F11.6 mm
Orientation	Transversal
Rotation	0.00 deg
A >> P	192 mm
R >> L	192 mm
F >> H	135 mm
Reset	Off

System - Tx/Rx

Frequency 1H	123.228035 MHz
Correction factor	1
Gain	High
Img. Scale Cor.	1.000
Reset	Off
? Ref. amplitude 1H	0.000 V

Physio - Signal1

1st Signal/Mode	None
TR	2060 ms
Concatenations	1

BOLD

GLM Statistics	Off
Dynamic t-maps	On
Ignore meas. at start	0
Ignore after transition	0
Model transition states	On
Temp. highpass filter	On
Threshold	4.00
Paradigm size	20
Meas[1]	Baseline
Meas[2]	Baseline
Meas[3]	Baseline
Meas[4]	Baseline
Meas[5]	Baseline
Meas[6]	Baseline
Meas[7]	Baseline
Meas[8]	Baseline
Meas[9]	Baseline
Meas[10]	Baseline
Meas[11]	Active
Meas[12]	Active
Meas[13]	Active
Meas[14]	Active
Meas[15]	Active
Meas[16]	Active
Meas[17]	Active
Meas[18]	Active
Meas[19]	Active
Meas[20]	Active
Motion correction	Off
Spatial filter	Off
Measurements	400
Delay in TR	0 ms
Multiple series	Off

Sequence - Part 1

Introduction	On
Multi-slice mode	Interleaved
Free echo spacing	Off
Echo spacing	0.64 ms
Bandwidth	1816 Hz/Px

Sequence - Part 2

EPI factor	64
RF pulse type	Normal
Gradient mode	Fast
