# Supplementary material for: Machine Learning-Based Identification of Suicidal Risk in Patients With Schizophrenia Using Multi-Level Resting-State fMRI Features
Source: Front Neurosci. 2021 Jan 11;14:605697. doi: 10.3389/fnins.2020.605697 (PMC7829970; doi:10.3389/fnins.2020.605697)

Supplementary Materials for

„Machine Learning based identification of suicidal risk in patients with schizophrenia using multi-level resting state fMRI features"

Bartosz Bohaterewicz, Anna M. Sobczak, Igor Podolak, Bartosz Wójcik, Dagmara Metel, Adrian, A. Chrobak, Magdalena Fąfrowicz, Marcin Siwek, Dominika Dudek, Tadeusz Marek

Machine Learning algorithms description:

Logistic regression

Logistic regression model for a binary problem gives a probability of $x$being in class C_1

$$p(C_1 | \varphi(x)) = \sigma(w^T \varphi(x)),$$

where $w$is a vector of parameters, and $\varphi(x)$is some transformation of input signal x, with $p(C_2 | \varphi(x)) = 1 - p(C_1 | \varphi(x))$(it can easily be extended to more classes). The function $\sigma(z) = 1/(1 + exp(-z))$maps computed internally $\sigma: R \ni z \rightarrow [0,1]$interval that can be interpreted as a probability value. Logistic regression is easily trained using a log-likelihood and gradient descent methods, that minimizes the cost function defined as

$$E(w) = -\ln\ p(t \mid w)\ =\ -\sum_{n=1}^{N} \{t_n \ln y_n + (1 - t_n) \ln(1 - t_n)\}.$$

Lasso

Simple logistic regression may exhibit some over-fitting behaviour, especially in cases where limited number of examples (in comparison to the number of parameters in w) are available. There are a number of regularization methods which reduce the size, and possibly the dimensionality, of the solution space. The most well known are the *weight decay*, frequently referred to as L2, method which keeps all parameters' values in restricted values, and a *lasso*, or L1, approach by modifying the cost function

$$E(w) = -\sum_{n=1}^{N} \{t_n ln\, y_n + (1 - t_n) ln(1 - t_n)\} + \frac{\lambda}{2} w^T w.$$

This has the effect of reducing all the elements of parameter vector $w$which are found *not*

to be important for the solution, of which some may be even removed, reducing the solution dimensionality (Hastie et al, 2008). This is very important in problems as here, where there is a small number of examples, but each of high dimensionality.

Support Vector Machine (SVM)

The support vector machines model is a *maximum margin classifier* methodology approach. SVMs are binary classifiers that root in a linear model

$$y = w^T \varphi(x) + b,$$

where is $\varphi(x): X \rightarrow F$is some transformation of the feature space F. If the problem represented by data is linearly separable, then there is an infinite number of solutions, i.e. hyperplanes $w$that separate examples from two classes. The SVM model selects an optimal dividing hyperplane that maximizes the so called *margin distance*

$$arg\ max_{w,b} \left\{ \frac{1}{||w||} min_n [t_n (w^T \varphi(x_n) + b)] \right\},$$

where $(x_n, t_n)$is an example pair, defined as the distance of a closest example to the hyperplane. It can be proven that such procedure maximizes the generalization by minimizing the chance that a new example would be incorrectly classified (Vapnik, 1995; Christiani and Shawe-Taylor, 2000; Scholkopf and Smola, 2002). As described above, SVM would be a simple extension of a binary classifier, save for the fact, that it is possible to define a *kernel function* $K(x, y) =< \varphi(x), \varphi(y) >$that returns distance $d(x, y)$in the feature space F*without* having to compute $\varphi(x)$explicitly. In case of the popular RBF kernel, the Fspace is of infinite dimension, which enhances the solution space, while the SVM still regularizes the solution. The implementation of an SVM might be complicated, although there are a number of well established libraries which we have also used.

Random Forest

Random forests are an example of an ensemble approach: build a number of models for a problem, each differing slightly from others. If we can ensure that the individual models are *diverse*, i.e. make errors in different parts of the data manifold, then we can guarantee that the eventual complex model selecting the final answer by some form of aggregation, e.g. averaging or voting, then it would have a higher accuracy rate than any individual one (Bishop, 2006; Hastie et al., 2008; Freund and Schapire, 1996). The models can even be said to be *weak*, i.e. have accuracy rates not much above a random classifier, provided they are still random.

Random forests are build of several decision trees (see e.g. Quinlan, 1986), all trained on overlapping subsets of original data. Each decision tree minimizes a cost function based on some cross-entropy of a Gini index trying to build a as shallow tree as possible, which helps the generalizability. After training even hundreds of separate trees, the overall accuracy raises visibly.

Since a decision tree checks some input data features separately in each tree node, it is possible to find a measure of importance of each of the features, which might not be possible with other models. Still, a preprocessing of data with a dimensionality reduction algorithm like PCA unfortunately prohibits the feature importance even in random forests.

Gradient Boosting

Gradient boosting is another example of algorithms built on the idea of ensemble and boosting models (Breiman). The procedure is an iterative one building a sequence of functions $F_k(x)$

$$F_k(x) = F_{k-1}(x) + arg\,min_h[\sum_{i=1}^{n} L(y_i, F_{k-1} + h_m(x_i))],$$

where h is some hypothesis selected at a step to minimize the error between the true output value and one predicted in the previous step. This is a popular approach to correct the error rather than to build one from scratch.

Description of the rsfMRI measures

3.1. ReHo

ReHo analysis depends on Kendall’s coefficient of concordance (Kendall & Gibbons, 1990), and was used to measure similarity of the time series of a given voxel and 26 neighbor voxels in voxel-by-voxel manner (Zang, Jiang, Lu, Tian, 2004). The ReHo was calculated on unsmoothed, filtered data and was smoothed with 4mm FWHM kernel afterwards. At the final step mReHo maps were obtained by dividing the mean ReHo of the whole brain within each voxel of the map.

3.2. ALFF

As the result of the low timescale of the hemodynamic response, BOLD signal is dominated by the low-frequency fluctuations (Bijsterbosch, Smith, Beckmann, 2017). The amplitude of the low-frequency fluctuations (ALFF) enables to estimate the total power within 0.01 - 0.08 Hz frequency range. ALFF was calculated on smoothed data without band-pass filter. At the final step mALFF maps were obtained by dividing the mean ALFF of the whole brain within each voxel of the map.

3.3. fALFF

Fractional amplitude of low-frequency fluctuations (fALFF) measures the power within a 0.01 - 0.08 Hz frequency range divided by the total power in the entire detectable frequency range (Zou et al., 2008). fALFF was calculated on smoothed data without band-pass filter. At the final step mfALFF maps were obtained by dividing the mean fALFF of the whole brain within each voxel of the map.

3.4. FC

Functional connectivity (FC) method indicates the temporal correlation of the Blood Oxygenation Level Dependent (BOLD) signal between functionally linked structures (Friston et al., 1993). The Pearson’s correlation coefficient was calculated based on the time-series extracted from two anatomically distinct brain regions (Li, Guo, Nie, Li & Liu, 2009). Correlation coefficients were transformed into z-scores using Fisher’s r-to-z for the purpose of normalization.

Table S1. ROIs used for intranetwork functional connectivity calculations.

Network	Abbreviation	Number of nodes
Dorsal Default Mode Network	dDMN	9
Ventral Default Mode Network	vDMN	10
Anterior Salience Network	aSN	7
Posterior Salience Network	pSN	12
Sensorimotor Network	SMN	6

Table S2. The hyperparameters learned by the grid search procedure for the five best classifiers

Model	Data	Result	Standardization	Dimensionality Reduction	Parameters
**LASSO**	FC Power 264 Static	70%/0.76	StandardScaler	None	alpha=0.001
**LR**	ALFF AAL 116 Dynamic	65%/0.75	MinMaxScaler	None	-
**GB**	fALFF AAL 116 Static	65%/0.74	StandardScaler	PCA number of components =3 (variance explained: 47.9%)	loss='deviance' learning_rate=0.1 n_estimators=100 max_depth=2 min_samples_split=2
**RF**	FC Power 264 Static	60%/0.69	MinMaxScaler	PCA number of components =15 (variance explained: 87.4%)	n_estimators=500 max_depth=None min_samples_split=0.5 max_features='log2'
**RF**	REHO Power 264 Static	55%/0.68	StandardScaler	PCA number of components =15 (variance explained: 91.1%)	n_estimators=500 max_depth=None min_samples_split=2 max_features='log2'

StandardScaler corresponds to removing the mean and scaling to unit variance described as z=(x-mu) / s independently for all the features.

MinMaxScaler scales and translates all individual independently features to be exactly within [0, 1] range: x_std = (x - min(x)) / (max(x) - min(x)).

Deviance is the binomial log-likelihood loss for binary classification returning the class probability

Fig. S1. box-and-whisker plot for three groups differences in total variance of dynamic functional connectivity for Power atlas

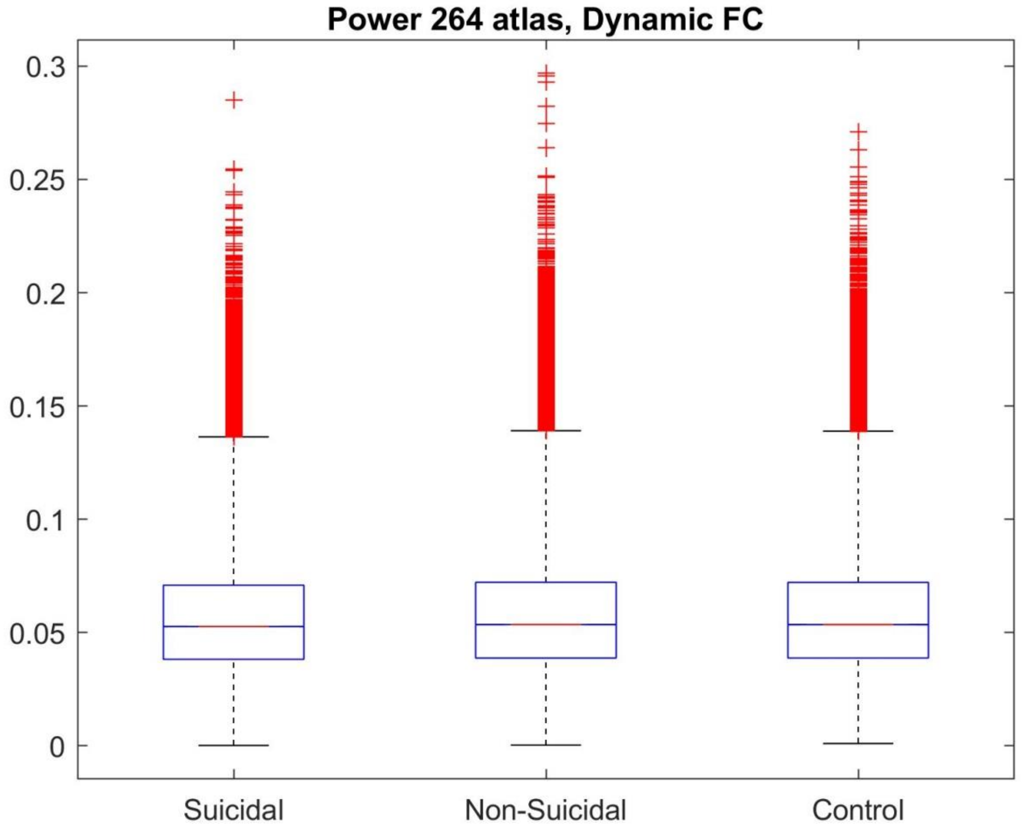

Fig. S2. . box-and-whisker plot for three groups differences in total variance of dynamic functional connectivity for AAL atlas

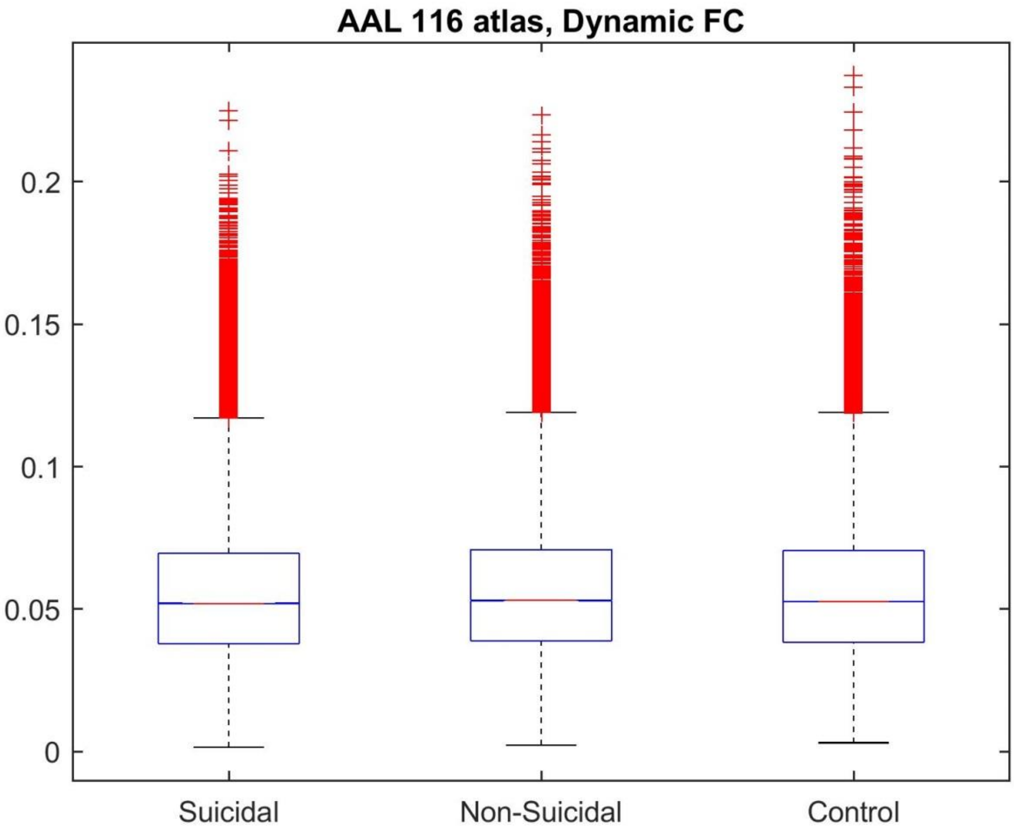

Supplement: Supplementary file 2 [file Data_Sheet_2.PDF]
